# Host cell cycle checkpoint as antiviral target for SARS-CoV-2 revealed by integrative transcriptome and proteome analyses

**DOI:** 10.1038/s41392-022-01296-1

**Published:** 2023-01-03

**Authors:** Liyan Sui, Letian Li, Yinghua Zhao, Yicheng Zhao, Pengfei Hao, Xuerui Guo, Wenfang Wang, Guoqing Wang, Chang Li, Quan Liu

**Affiliations:** 1 Department of Infectious Diseases, Infectious diseases and Pathogen Biology Center, Key Laboratory of Organ Regeneration and Transplantation of the Ministry of Education, The First Hospital of Jilin University, State Key Laboratory of Zoonotic Diseases, Changchun, 130021 China; 2 Research Unit of Key Technologies for Prevention and Control of Virus Zoonoses, Chinese Academy of Medical Sciences, Changchun Veterinary Research Institute, Chinese Academy of Agricultural Sciences, Changchun, 130117 China; 3 College of Basic Medical Science, Jilin University, Changchun, 130021 China

**Keywords:** Microbiology, Infection

**Dear Editor**,

Severe acute respiratory syndrome coronavirus 2 (SARS-CoV-2), the causative agent of the COVID-19 pandemic, has posed severe threats to global public health, highlighting an urgent need to understand its pathogenesis and to develop antiviral therapies. Both DNA and RNA viruses can modulate cell cycle progression to maximize their replication.^1^ However, the effects of SARS-CoV-2 on cell cycle progression remains largely unknown.

Here, in our efforts to identify host factors associated with SARS-CoV-2 infection by proteome analysis (Supplemental Figs. S1 and S2), cell cycle-related proteins were found to be the most enriched proteins upon SARS-CoV-2 infection at both 12 and 24 h post-infection (hpi) (Supplemental Fig. S2c, d). In detail, expressions of the regulators of cell cycle, including cyclin-dependent kinase 1 (CDK1), CDK2, cyclin B1, and other cell cycle-related proteins, such as cell division cycle 20 (Cdc20), Wee1-like protein kinase (WEE1), Bub1, Bub3, and aurora kinase A (AURKA) were all increased at 12 hpi, while decreased at 24 hpi (Fig. 1a and Supplemental Fig. S3a–e). Immunoblots further confirmed the expression of cyclin B1, CDK1 and CDK2 upon SARS-CoV-2 infection (Fig. 1b and Supplemental Fig. S3f-i). Transcriptome analysis also showed an enrichment of cell cycle-related transcripts at 24 hpi (Supplemental Fig. S2e, f), and the profile of CDK2, cyclin B1, WEE1, and AURKA at the mRNA expression levels was consistent with the protein levels (Fig. 1a and Supplemental Fig. S3a, e). Overall, the integrative transcriptome and proteome analyses indicated that SARS-CoV-2 infection may interfere with host cell cycle progression.

**Fig. 1:**
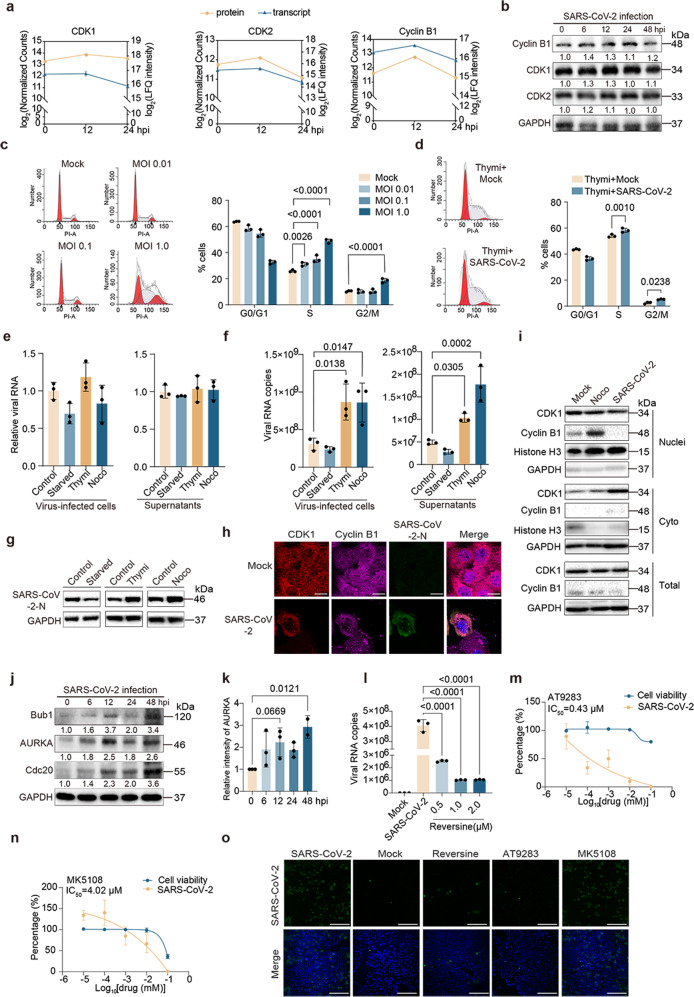
Transcriptome and proteome reveals host cell cycle checkpoint as antiviral target for SARS-CoV-2. **a** Log_2_ fold change profiles of mRNA and protein levels of CDK1 (Cyclin-dependent kinase 1), CDK2 and cyclin B1 in Caco-2 cells infected with SARS-CoV-2. **b** Caco-2 cells were infected with SARS-CoV-2 at the MOI of 0.01. The cells were collected at 0, 6, 12, 24, and 48 hpi and the indicated proteins were analyzed by immunoblots. **c** Caco-2 cells were mock-infected or infected with SARS-CoV-2 at the MOI of 0.01, 0.1 and 1.0, respectively. After 48 h, cells were harvested and the cell cycle was analyzed by flow cytometry. Three independent experiments were conducted, and the data were shown in the column graphs. **d** Caco-2 cells synchronized to the S phase by 0.85 mM thymidine (Thymi) were mock-infected or infected with SARS-CoV-2 at the MOI of 0.1. After 48 h, cells were collected to analyze the cell cycle by flow cytometry. Three independent experiments were conducted, and the data were shown in the column graph. **e**–**g** Caco-2 cells were grown in a medium with no serum, 0.85 mM Thymi, or 50 ng/ml nocodazole (Noco) to block cells in the G0/G1, G2/M and S phases, the cells were then mock-infected or infected with SARS-CoV-2 at the MOI of 0.1, cells and supernatants were harvested after 30 min (**e**) or 48 h (**f**, **g**). The mRNA level of SARS-CoV-2 nucleocapsid (N) was examined by qPCR (**e, f**), and the protein level of SARS-CoV-2 N was analyzed by immunoblot (**g**). **h** Caco-2 cells were mock-infected or infected with SARS-CoV-2 at the MOI of 0.01. After 48 h, the cells were fixed and stained with indicated antibodies. Red, CDK1 signal; Pink, cyclin B1 signal; Green, SARS-CoV-2 N signal; Blue, DAPI (the nuclear signal). Bar, 10 μm. **i** Caco-2 cells were mock-infected, infected with SARS-CoV-2 at the MOI of 0.01, or treated with 50 ng/ml nocodazole. After 48 h, the cells were harvested and the separated nuclear and cytoplasmic proteins were analyzed for cyclin B1 and CDK1 by immunoblot. **j**, **k** Caco-2 cells were infected with SARS-CoV-2 at the MOI of 0.01, cells were collected at 0, 6, 12, 24 and 48 hpi, and the indicated proteins were analyzed by immunoblot (**j**). Gray-scale statistical analysis of AURKA protein was examined by immunoblot using the Image J (**k**). **l**–**n** Caco-2 cells were mock-infected or infected with SARS-CoV-2 at the MOI of 0.01 and treated with the indicated concentrations of reversine (**l**), AT9298 (**m**) and MK5108 (**n**), the supernatants were collected at 48 hpi and viral copies was examined using qPCR. **o** Caco-2 cells were mock-infected or infected with SARS-CoV-2 at the MOI of 0.01 and treated with 2.0 μM reversine, 10 μM AT9298, 10 μM MK5108 or DMSO. The cells were fixed at 48 hpi and stained for the indicated proteins. Green: SARS-CoV-2 N protein signal; Blue, DAPI (the nuclear signal). Bar, 100 μm

Next, we determined if SARS-CoV-2 infection manipulates host cell cycle progression. Caco-2 cells infected with SARS-CoV-2 were analyzed for cell cycle distribution by flow cytometry, revealing that there were 26.1% of the mock-infected cells in the S phase, while 31.1%, 35.1%, and 48.5% of cells in the S phase after SARS-CoV-2 infection at the multiplicity of infection (MOI) of 0.01, 0.1 and 1.0, respectively, with a significant difference between the viral infection groups and control group (Fig. 1c). An obvious accumulation of cells in the G2/M phase was also observed in SARS-CoV-2-infected cells (19.5%, at the MOI of 1.0) as comparison with the mock-infected cells (10.3%) (Fig. 1c). Furthermore, Vero and HEK293T-hACE2 cells (293T cells expressing human angiotensin-converting enzyme 2) also displayed significantly higher cellular proportions of S and G2/M phases after SARS-CoV-2 infection (Supplemental Fig. S4a–d). The viral infection was shown to affect the S phase progression at early infection stage and to interfere with the G2/M phase progression at the late infection stage (Supplemental Fig. S4e, f). Taken together, these results indicated that SARS-CoV-2 manipulates the host cell cycle and causes cell cycle arrest at the S and G2/M phases.

Given that cells in different cell-cycle phases may affect the virus-mediated cell cycle arrest, we further synchronized cell populations to confirm the effect of SARS-CoV-2 infection on cell cycle progression. Caco-2 cells were synchronized to the G0/G1, S, and G2/M phases using serum starvation, thymidine, or nocodazole, respectively (Supplemental Fig. S5a, b). For the thymine-treated cells, the percentage of S and G2/M cells was 54.0% and 2.5% in the mock-infected group, while they were increased to 58.2% and 5.2% in the SARS-CoV-2 infection group, which was significantly higher than the mock infection group (Fig. 1d). SARS-CoV-2 infection of serum deprivation and nocodazole-treated cells also resulted in significantly higher proportions of S and G2/M phases (Supplemental Fig. S5c, d), confirming that SARS-CoV-2 infection can induce cell cycle arrest at the S and G2/M phases.

Viruses manipulate cell cycle progression to generate resources and cellular conditions beneficial for viral assembly and replication.^2^ We thus detected if synchronization of G2/M and S-phases can promote viral replication. After blocked at different phases, Caco-2 cells were infected with SARS-CoV-2. At 30 min post-infection, there was no obvious difference in viral replication between the control and synchronized cells (Fig. 1e). However, at 48 hpi, SARS-CoV-2 replicated significantly higher in the cells treated with thymine and nocodazole, while the viral replication was suppressed in the starvation group (Fig. 1f, g), indicating that synchronization of the G2/M- and S-phases does not affect viral adsorption, but promotes the replication of SARS-CoV-2.

We further investigated the effects of SARS-CoV-2 infection on cell cycle regulators of S/G2 phases, showing that the abundance of cyclin B1, CDK1 and CDK2 was increased before 24 hpi, but decreased at 48 hpi (Fig. 1b and Supplemental Fig. S3f–i). We also examined the nuclear translocation of cyclin B1 and CDK1, which is critical for cells to enter the mitotic phase.^3^ Immunofluorescence showed interrupted nuclear translocation of cylinB1 and CDK1, and nuclear fractionation analysis revealed reduced cyclin B1 and CDK1 in the nuclei after SARS-CoV-2 infection (Fig. 1h, i).

During cell cycle progression, cyclin B1 can be accumulated in the G2 phase, whose ubiquitination and degradation through anaphase-promoting complex (APC/C) is essential for cells to exit mitosis. APC/C can be manipulated by viruses to induce cells arrest at the G2/M phase.^4^ The activity of APC/C can be regulated by Cdc20 and the spindle assembly checkpoint (SAC).^5^ As seen in the proteomic profiling of SARS-CoV-2 infected cells, the expression of two SAC members (Bub1 and Bub3) and Cdc20 was elevated at 12 hpi, while decreased at 24 hpi (Supplemental Fig. S3b–d). Similarly, there were 21 increased APC/C substrates, whereas 4 decreased substrates upon SARS-CoV-2 infection at 12 hpi (Supplemental Fig. S6a and Table S1). Immunoblot confirmed that the expression levels of Bub1, Cdc20, and AURKA (substrate of APC/C) were elevated at 12 hpi, while decreased at 24 hpi, and displayed another elevation at 48 hpi (Fig. 1j, k, and Supplemental Fig. S6b, c), indicating that SARS-CoV-2 infection can suppress the activity of APC/C at 24 hpi.

To evaluate the contribution of SAC activation to SARS-CoV-2 replication, we tested the antiviral effect of the inhibitor, reversine, a pan inhibitor of aurora kinases, which can specifically inhibit the SAC. Reversine inhibited half-cell viability at the concentration of 2.25 μM (Supplemental Fig. S7), thus, 0.5 μM, 1.0 μM, and 2.0 μM of reversine was used to test its inhibitory effect on replication of SARS-CoV-2, showing that 0.5 μM reversine had an inhibitory rate of 93.8% for viral replication, 1.0 μM and 2.0 μM reversine had an inhibitory rate of 97.4% as comparison with DMSO-treated group (Fig. 1l).

Moreover, as the expression of AURKA was significantly increased upon SARS-CoV-2 infection at 48 hpi (Fig. 1k), we also employed two inhibitors of AURKA, AT9283 and MK5108, to test their inhibitory effects on SARS-CoV-2 replication, showing that 0.01 μM AT9283 had no significant effect on viral replication, 0.1 μM and 1 μM AT9283 had an inhibitory rate of 50.0–66.2% for viral replication, 10 μM and 100 μM AT9283 had an inhibitory rate of 90.1–99.7% as comparison with the control group, with the half maximal inhibitory concentration (IC_50_) value of 0.43 μM in Caco-2 cells (Fig. 1m); while 0.01 μM, 0.1 μM and 1 μM MK5108 showed no significant inhibitory effect on replication of SARS-CoV-2, 10 μM MK5108 had an inhibitory rate of 33.2%, and 100 μM MK5108 had an inhibitory rate of 99.1%, with the IC_50_ value of 4.02 μM (Fig. 1n). Consistently, immunofluorescence analysis showed that reversine and AT9283 significantly suppressed the replication of SARS-CoV-2 (Fig. 1o). These data indicated that inhibitors of SAC and AURKA can effectively suppress SARS-CoV-2 replication in vitro.

In summary, our findings reveal that SARS-CoV-2 manipulates cell cycle checkpoint and induces host cells arrest at the G2/M and S phases to facilitate viral replication, and inhibitors of SAC and AURKA can effectively inhibit viral replication, suggesting a potential antiviral target of host cell cycle checkpoint for COVID-19 (Extended Discussion).

## Supplementary information

Supplemental material

Supplemental material

## Data Availability

The mass spectrometry data were uploaded to iProX (IPX0003647000) and the raw transcriptome data were uploaded to the NCBI Sequence Read Archive (PRJNA783650).
