# Supplementary material for: Host cell cycle checkpoint as antiviral target for SARS-CoV-2 revealed by integrative transcriptome and proteome analyses
Source: Signal Transduct Target Ther. 2023 Jan 3;8:21. doi: 10.1038/s41392-022-01296-1 (PMC9808731; doi:10.1038/s41392-022-01296-1)
Supplement: Supplementary file 1 — Supplemental material [file 41392_2022_1296_MOESM1_ESM.docx]

**Supplementary Information for**

Host cell cycle checkpoint as antiviral target for SARS-CoV-2 revealed by integrative transcriptome and proteome analyses

Liyan Sui^1^, Letian Li^2^ , Yinghua Zhao^1^, Yicheng Zhao^3^, Pengfei Hao^2^, Xuerui Guo^4^, Wenfang Wang^5^, Guoqing Wang^5*^, Chang Li^2*^, Quan Liu^1,6*^

Correspondence: Guoqing Wang ([qing@jlu.edu.cn](mailto:qing@jlu.edu.cn)), Chang Li (lichang78@163.com) or Quan Liu (liuquan1973@hotmail.com)

**This PDF file includes:**

Materials and Methods

Supplementary Figures S1 to S8

Extended Discussion

**MATERIAL AND METHODS**

**Cells and virus**

Caco-2 cells, HEK293T-hACE2 (293T cells expressing human angiotensin-converting enzyme 2), and Vero E6 cells were cultured in DMEM (Invitrogen, Carlsbad, USA) containing 1% penicillin-streptomycin and 10% FBS (Gibco, Gaithesburger, USA) at 37 °C in a 5% CO_2_ humidified atmosphere. HEK293T-hACE2 cells were kindly provided by Zhong Ji Dang Kang Biotechnology Co., Ltd., Beijing, China. All cells were tested for mycoplasma-free. The SARS-CoV-2 virus (GenBank access number MT291831.1) originated from human throat swabs were propagated in Vero E6 cells in fresh medium supplemented with 2% FBS and titred using a tissue culture median infectious dose assay (TCID_50_).

**Antibodies and drugs**

Anti-CDK1, anti-CyclinB1, anti-Bub1, anti-Cdc20, anti-AURKA, anti-H3 and anti-GAPDH antibodies, CoraLite594-conjugated goat anti‐rabbit IgG and CoraLite488-conjugated Goat Anti-Rabbit IgG were obtained from Proteintech (Wuhan, China); anti-SARS-CoV and its NP antibodies were purchased from ZENBIO (Chengdu, China), the detailed information about antibodies were listed in Supplemental Table S4. Nocodazole, thymidine and reversine were purchased from Selleck (Houston, USA).

**Infected cells for proteome and transcriptome**

Caco-2 cells seeded in 25 cm^2^ tissue-culture flasks (Corning, California, USA) were allowed to attach for 24 h, and inoculated with SARS-CoV-2 at a multiplicity of infection (MOI) of 0.01 in serum-free DMEM for 1 h at 37°C. The infected samples were harvested immediately after absorption (0 h), or at 12 h and 24 h post infection (hpi) for further analysis.

**Proteome analysis**

The SARS-CoV-2-infected Caco-2 cell samples (0, 12, and 24 hpi) were lysed by sonication after being frozen at -80°C and centrifuged at 12,000 g for 10 min at 4°C to remove cell debris. The supernatants were transferred to a new centrifuge tube, and the protein concentration was determined using a BCA kit (Thermo, Waltham, USA). An equal amount of protein from each sample was acetone precipitated and subjected to trypsin digestion for mass spectrometry analysis to determine total protein abundance.

Liquid chromatography-mass spectrometry (LC-MS) based proteomics was conducted by PTM BioLab Co., Ltd., Hangzhou, China. Briefly, the peptides were separated using the NanoElute system (Bruker) and separated by an ultra-high-performance liquid phase system, which was injected into a Capillary ion source for ionization and then analyzed by timsTOF Pro mass spectrometer (Bruker). The peptide precursor ions and their secondary fragments were detected and analyzed using high-resolution TOF using Parallel Accumulation Serial Fragmentation (PASEF) mode.

**Transcriptome analysis**

Caco-2 cells infected with SARS-CoV-2 were harvested at indicated time points (0, 12, and 24 hpi). Total RNA was extracted by Trizol (Invitrogen, Carlsbad, USA), and RNA-seq libraries were prepared according to standard protocol ^1^. Libraries were sequenced with Illumina HiSeq^TM^ 4000 by Guangzhou Gene Denovo Biotechnology Co. Ltd., China. Transcripts were quantified and annotated with feature counts (v1.6.0). All transcripts were normalized and differentially analyzed in DESeq2.

**Bioinformatics analysis**

The raw proteomics data were processed by LFQ (label-free quantification) in MaxQuant (version 1.6.17.0). The data were then processed using MSstats with default values, and the *P* value was calculated using Student’s t-test and corrected by Benjamini-Hochberg (BH) method. Differential protein expression analysis by principal components analysis (PCA) and volcano plots for 0, 12 and 24 hpi samples were processed using DESeq2 and ggplot2, we identified as log2 fold change > 1 as up-regulated proteins/mRNA and log2 fold change < -1 as down-regulated proteins/mRNA (*P* <0.05). The enrichment analysis of gene ontology (GO) and KEGG pathways was performed via ClueGo or cluster Profiler package in R (4.0.4) with default parameters.

**Cell cycle analysis by flow cytometry**

Cells were fixed in 70% ethanol overnight at 4℃, then washed with PBS and stained with PI/RNase Staining Buffer Solution (BD Biosciences, California, USA) for 15 min in the dark. The cell cycle distribution was analyzed using a BD LSRFortessa flow cytometer. Data analysis was carried out using ModFit LT 5.0 (Verity Software House, Maine, USA).

**Cell cycle** **synchronization**

Caco-2 cells were plated in a 12-well plate to attach overnight. Then the media was replaced with DMEM without serum to induce G0/G1 arrest. For S-phase synchronization, the cells were treated with 0.85 mM thymidine. For G2/M arrest, cells were treated with 50 ng/μL nocodazol. The synchronized cells were then inoculated with the virus for 1 h and cultured in a fresh medium for 48 h.

**Immunoblot analysis**

Immunoblot analysis was performed as described elsewhere.^2^ Briefly, cells were collected and resuspended in lysis buffer with TM protease inhibitors (Selleck, Houston, USA) and incubated on ice for 30 min. The cell lysis was then centrifuged at 12,000 × g for 15 min, and the supernatants were collected and transferred to PVDF membrane by 12% SDS-PAGE gel electrophoresis (Millipore, MA, USA). Blots membrane was blocked and incubated with the indicated antibodies listed in Supplemental Table S4. The membranes were tested using ECL (Thermo, Waltham, USA) and protein bands were visualized by Biorad CHemiDoc XRS (Biorad, California, USA).

To detect the nuclear translocation of cylinB1 and CDK1, the nuclear and cytoplasmic proteins of the indicated cells were first separated by the nuclear and cytoplasmic protein extraction kit (Beyotime, China) according to the manufacturer’s instructions. The purified cytoplasmic and nuclear fractions were subjected to immunoblot with the relevant antibodies.

**Immunofluorescence analysis (IFA)**
An immunofluorescence assay was carried out to assess the nuclear translocation of cyclinB1 and CDK1 ^3^. Cells were fixed with 4% paraformaldehyde for 30 min and permeabilized with 1% Triton X-100. After blocking with the blocking buffer (PBS containing 1% bovine serum albumin), cells were incubated with primary and secondary antibodies and stained for nuclear. Fluorescence was captured on OLYMPUS FV3000 confocal microscope (Olympus, Shinjuku, Japan).

**Cell viability assay**

Caco-2 cells were plated into 96-well plates and left adherence for 24 h. The culture medium was then discarded, and drugs/compounds (0-100 μM) mixed in 100 μL DMEM containing 2% FBS were added into the plates. After incubation for 48 h, 10 μL of CCK8 solution (Dojindo Laboratories, Kumamoto, Japan) was added to each well, and the OD value at 450 nm was measured after another 4 h of incubation.

**Viral inhibition assay**

Caco-2 cells were seeded in 96-well plates at 5,000 cells/well. After the adherence of cells, SARS-COV-2 (MOI of 0.01) was added to infection with indicated concentration of drugs or DMSO as control. Each concentration of drugs was conducted in three biologically replicates. After 48 h, the supernatants were collected for virus quantification by qPCR.

**Quantitative real-time RT-PCR (qPCR)**

RNA from the infected host cells and the supernatant were extracted by the UNlQ-10 Column Trizol total RNA isolation kit (Sangon, Shanghai, China), and cDNA was synthesized using TransScript^®^ One-Step cDNA Synthesis SuperMix (TransGen, Beijing, China). Virus copies and gene expression were determined by the HiScript II One Step qRT-PCR SYBR Green Kit (Vazyme, Nanjing, China) using the primers listed in Supplemental Table S5.

**Statistical analysis**

Statistical analysis was performed using GraphPad Prism, and data were shown as the mean ± SD values. Two-tailed unpaired Student’s t-tests were used for two-group comparisons, and the P value of less than 0.05 was considered statistically significant.


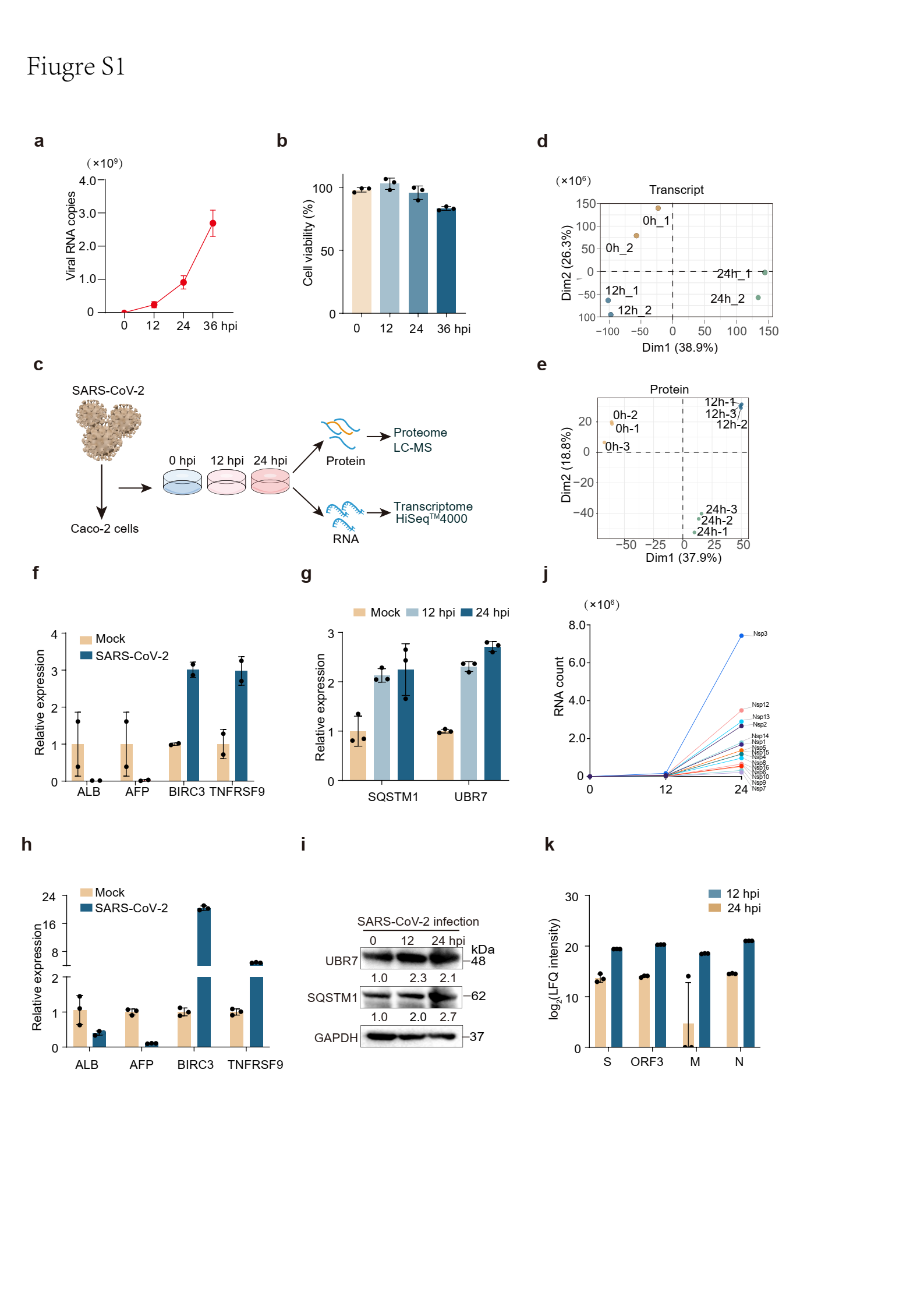


**Fig. S1** Changes of host and viral transcripts and proteins after SARS-CoV-2 infection. **a, b** To investigate how SARS-CoV-2 regulates host cellular signaling, we first determined the viral infection conditions in Caco-2 cells, a human colorectal adenocarcinoma cell line susceptible to SARS-CoV-2 infection.^4^ Caco-2 cells infected with SARS-CoV-2 at an MOI (multiplicity of infection) of 0.01 were harvested at 0, 12, 24 and 36 hours post infection (hpi). The N gene of SARS-CoV-2 was detected by qPCR and cell viability was examined by cell counting Kit-8 (CCK-8) assay. **c** Transcriptome and proteome analysis workflow of SARS-CoV-2-infected cells. Caco-2 cells infected with SARS-CoV-2 were harvested at 0, 12 and 24 hpi in biological duplicates for transcriptome and triplicates for quantitative proteome analysis. d, e Principal component analysis (PCA) of transcriptome (**d**) and proteome (**e**). PCA was conducted with the R package (factoextra). Sequencing reads or protein intensities were log_2_ transformed, followed by PCA and visualization. **f, g** The results of transcriptome and proteome were verified by qPCR and immunoblot assay. mRNA expression changes of Afamin (ALB), Alpha-fetoprotein (AFP), Baculoviral IAP repeat-containing protein 3(BIRC3) and Tumor necrosis factor receptor superfamily member 9 (TNFRSF9) (**f**) and protein expression changes of UBR7 and Sequestosome-1 (SQSTM1) (**g**) in transcriptome and proteome data. **h** qPCR analysis of the *ALB, AFP, BIRC3* and *TNFRSF9* in SARS-CoV-2 infected cells at 24 hpi, GAPDH was used as control. **f** Immunoblot analysis of SQSTM1 and UBR7 in SARS-CoV-2 infected cells at 12 and 24 hpi. **j, k** Successful infection was confirmed by increasing abundance of the viral proteins. Transcription (**j**) and translation (**k**) of viral proteins during the course of infection. nsp: non-structural proteins; S: spike protein; ORF3: open reading frame 3; M: membrane protein; N: nucleocapsid protein.


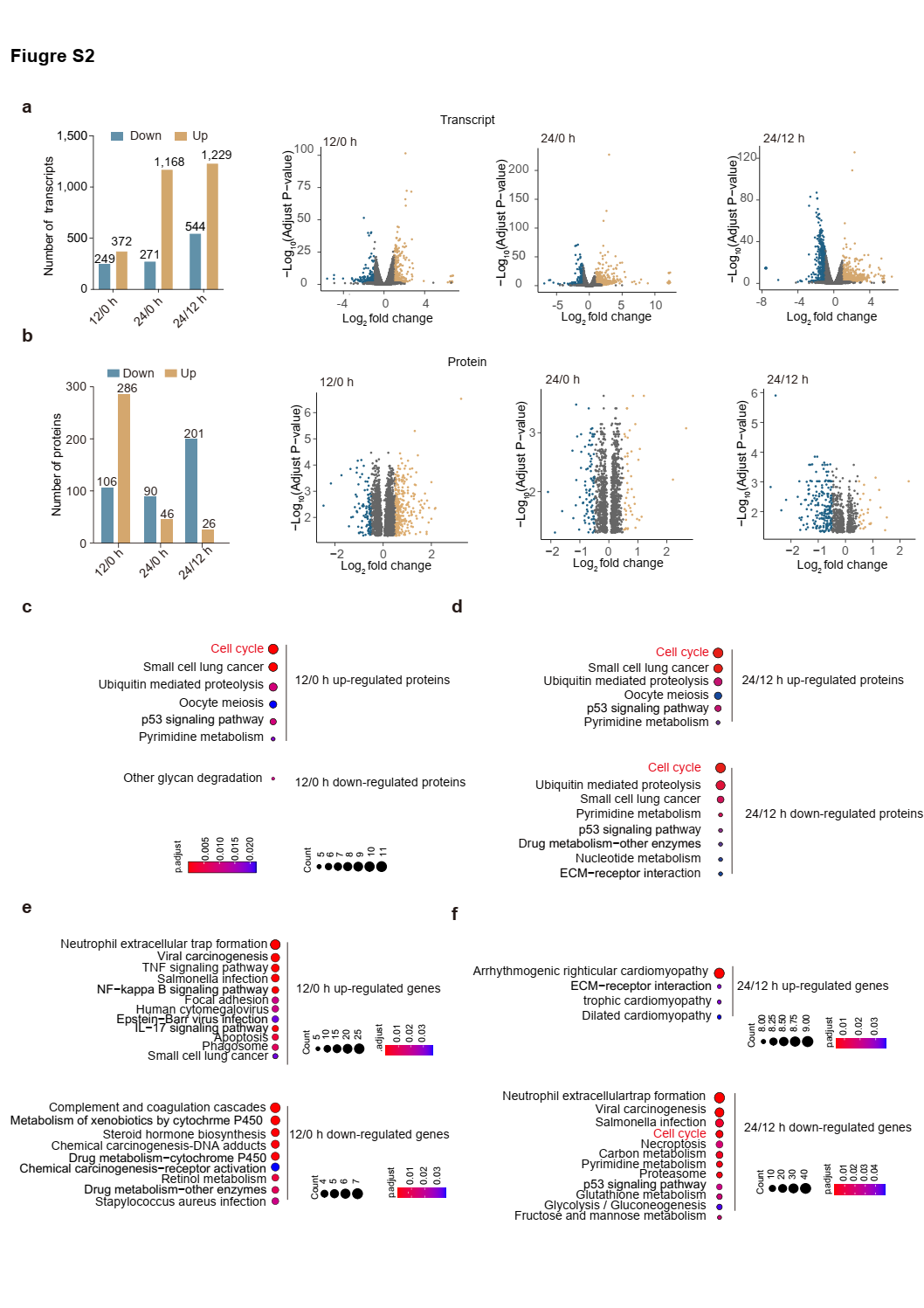


**Fig. S2** Volcano plot of proteomic and transcriptional data and KEGG analysis of the significantly changes proteins and genes. **a,b** The number of significantly changed host transcripts (**a**) and proteins (**b**) upon SARS-CoV-2 infection and Volcano plot of transcript and protein expression in cells. **c, d** KEGG (Kyoto encyclopedia of genes and genomes) analysis of the up- and down-expressed proteins at 12 and 24 hpi. **e, f** KEGG analysis of the up- and down-expressed genes at 12 and 24 hpi. Depth of color indicates the significance of the term (adjusted P-value), the size indicates the counts of genes.


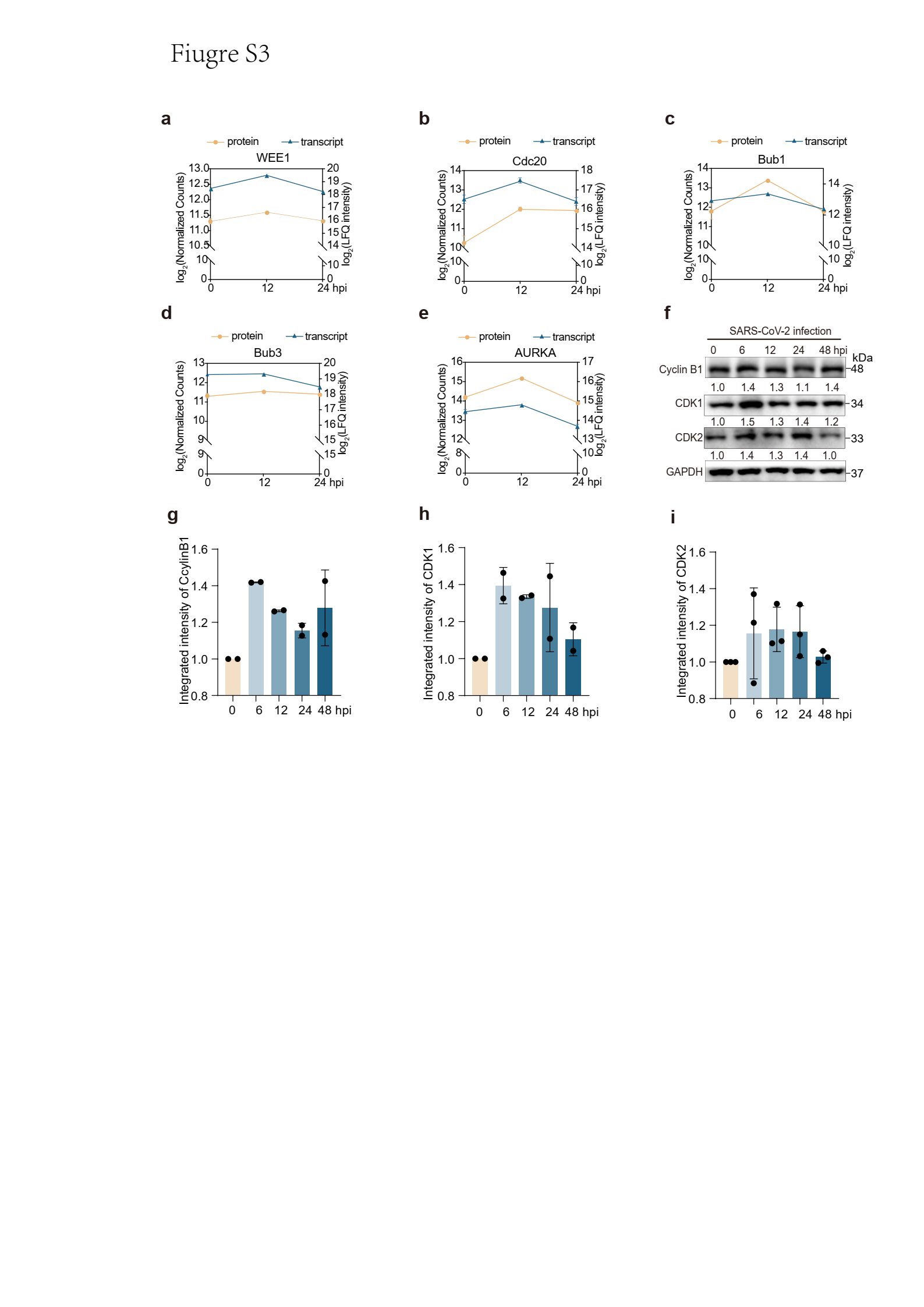


**Fig. S3** Changes of cell-cycle related proteins during SARS-CoV-2 infection. **a-e** Log_2_ fold change profiles of mRNA and proteins levels of Wee1-like protein kinase (WEE1) (**a**), cell division cycle 20 (Cdc20) (**b**), Bub1(**c**), Bub3 (**d**) and aurora kinase A (AURKA) (**e**) during SARS-CoV-2 infection in Caco-2 cells. **f** Caco-2 cells were infected with SARS-CoV-2 at an MOI of 0.01, cells were collected at 0, 6, 12, 24 and 48 hpi, and the indicated proteins were analyzed by immunoblot. **g-i** Gray-scale statistical analysis of CDKs and cyclin B1 protein examined by immunoblot using the Image J.


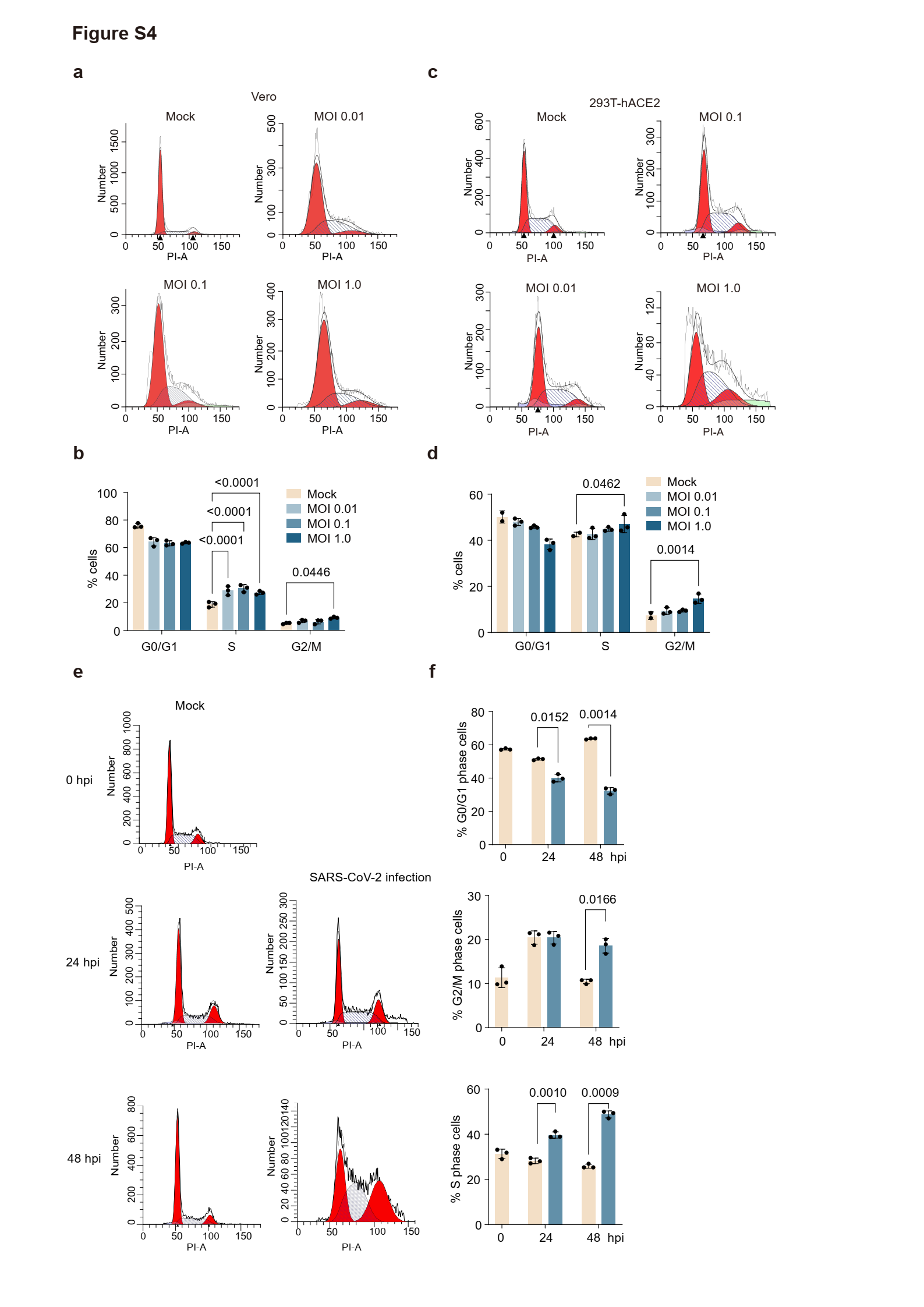


**Fig. S4** SARS-CoV-2 infection induces cell cycle arrest at the G2/M and S phases. **a-d** Vero (**a**) and 293T-ACE2 cells (**c**) were mock-infected or infected with SARS-CoV-2 at an MOI of 0.01, 0.1 and 1.0, respectively. After 48 h, cells were harvested and the cell cycle was analyzed by flow cytometry. Three independent experiments were conducted, and the data were shown in column graphs for Vero (**b**) and 293T-ACE2 (**d**). **e, f** Caco-2 cells were mock-infected or infected with SARS-CoV-2 of a MOI of 1.0. After 24 and 48 h, cells were harvested, and the cell cycle distribution was analyzed by flow cytometry. Three independent experiments were conducted, and the data were showed in column graph.


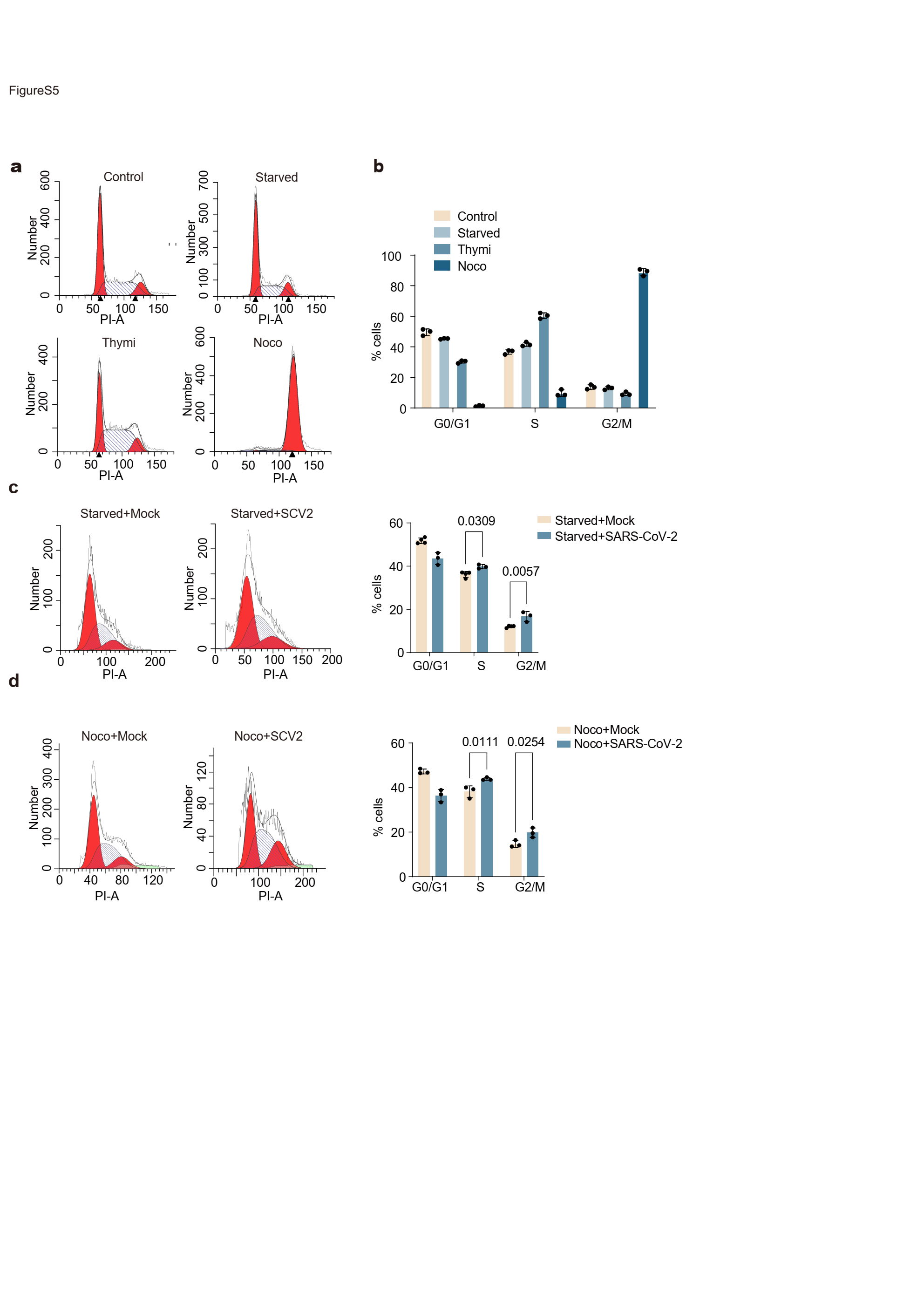


**Fig. S5** Synchronization of the G2/M and S phases promotes SARS-CoV-2 replication in infected cells. **a**, **b** Caco-2 cells were grown in a medium with serum starvation, 0.85 mM thymidine (Thymi), or 50 ng/ml nocodazole (Noco) to block cells in the G0/G1, G2/M and S phases. The cell cycle was analyzed by flow cytometry (**a**) and the data were shown in column graph (**b**). **c, d** Caco-2 cells synchronized to G0/G1 and G2/M phases by serum starvation (**c**) and 50 ng/ml Noco (**d**) were mock-infected or infected with SARS-CoV-2 at an MOI of 0.1. After 48 h, cells were collected, and the cell cycle was analyzed by flow cytometry. Three independent experiments were conducted, and the data were shown in a column graph.


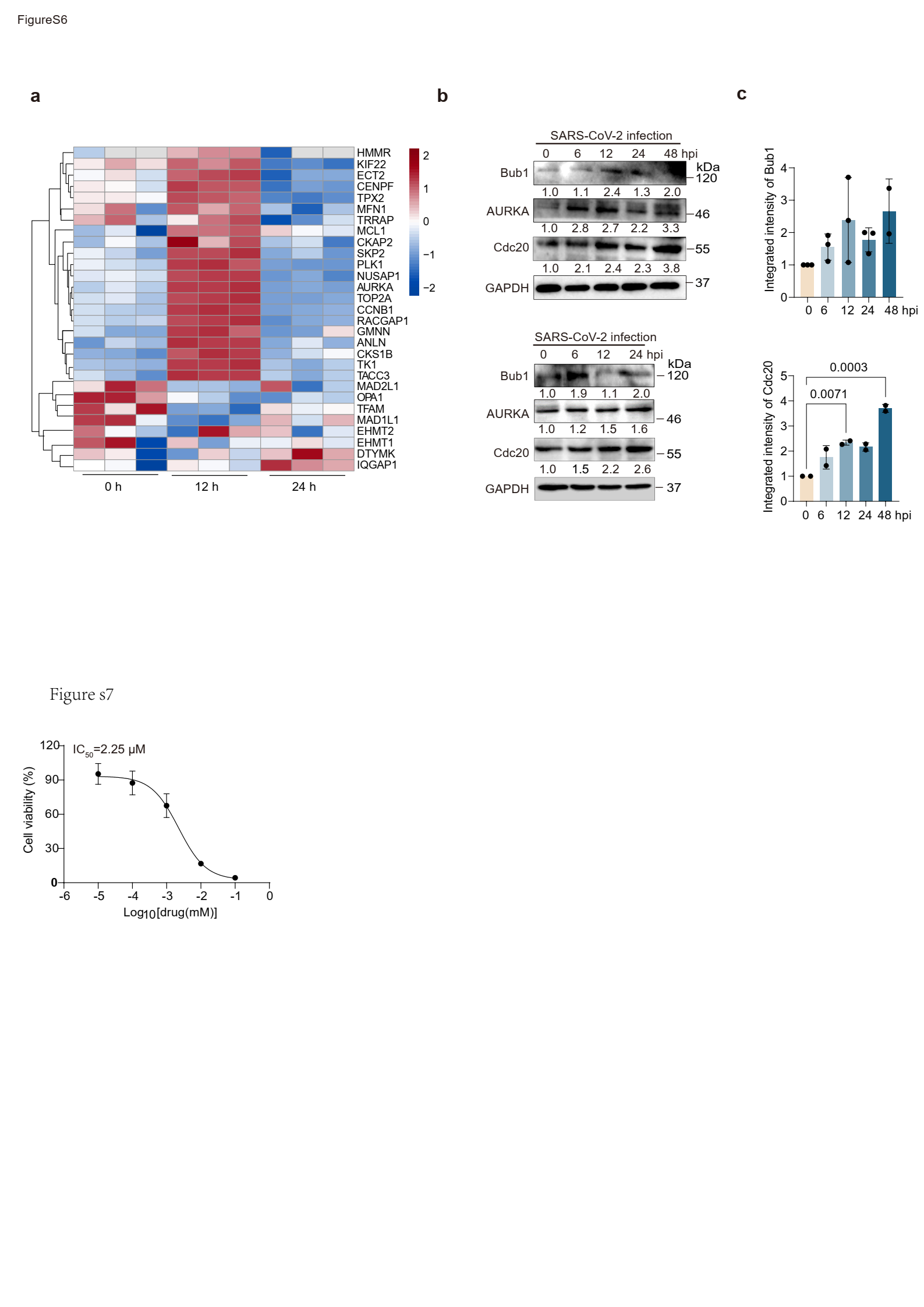


**Fig. S6** Changes of protein level of Bub1, AURKA and Cdc20 during SARS-CoV-2 infection. **a** Heatmap of the relative expression levels of the substrates of APC/C upon SARS-CoV-2 infection. **b** Caco-2 cells were infected with SARS-CoV-2 at an MOI of 0.01, cells were collected at 0, 6, 12, 24 and 48 hpi, and the indicated proteins were analyzed by immunoblot. **c** Gray-scale statistical analysis of Cdc20 and Bub1 protein examined by immunoblot using the Image J.


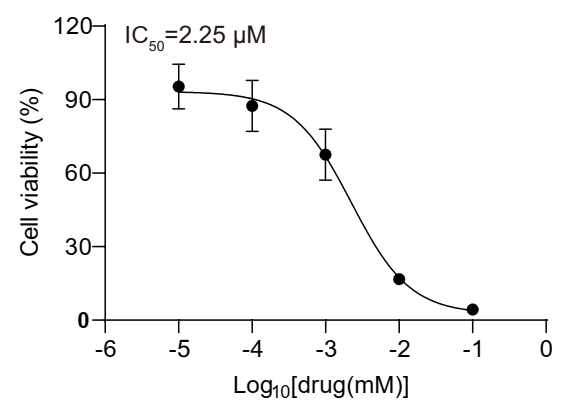


**Fig. S7** Half-maximum inhibitory concentration (IC50) value of reversine to Caco-2 cells computed from the curve fit.


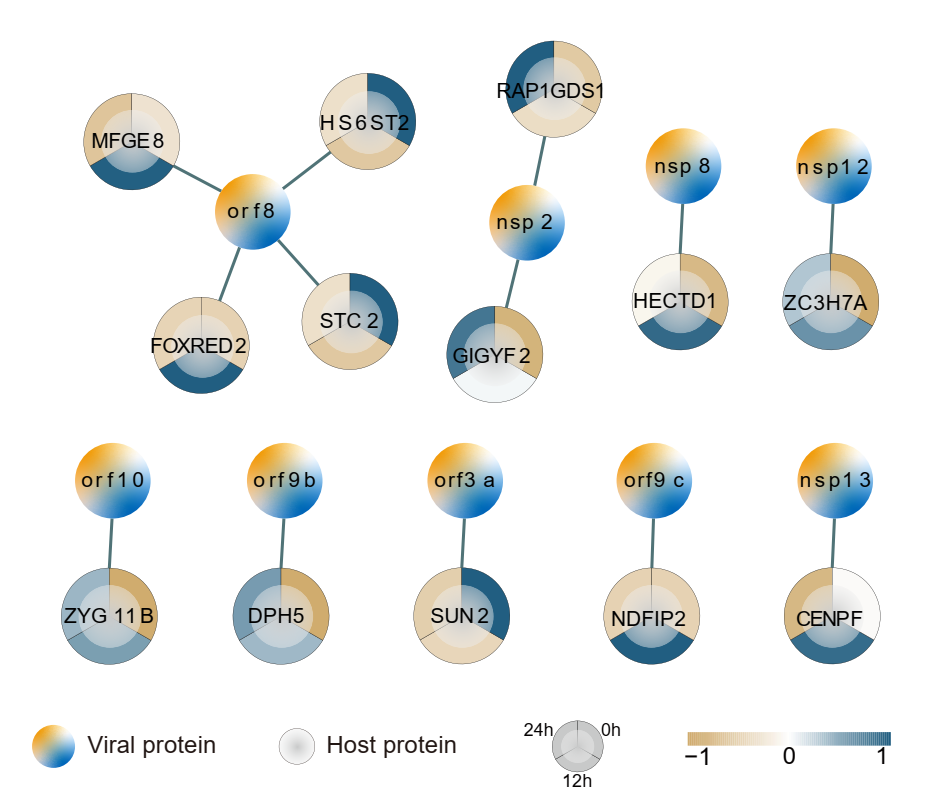


**Fig. S8** Interaction between viral and host proteins in infected cells. The significantly dysregulated host proteins upon SARS-CoV-2 infection interacted with viral proteins.^5^ Viral proteins were shown as small camouflage balls. Interacting host proteins were shown as white balls, colored by their z-score fold changes compared with 0 h infected samples (blue, increase; yellow, decrease) at 0, 12, and 24 hpi.

**Extended Discussion:**

Dysregulating the cell cycle is a common strategy employed by both DNA and RNA viruses to exploit the host cell machinery for their own benefit. The RNA virus enterovirus D68 can manipulate host cell cycle arrest at the G0/G1 phase,^6^ while the Dabie bandavirus induces G2 cell cycle arrest to promote viral replication.^7^ The DNA virus hepatitis B virus (HBV) blocks the cell cycle at the G1 phase to facilitate viral accumulation.^8^ Our study confirmed that SARS-CoV-2 infection induced cells blocked at G2/M and S phases, which was consistent with the previous report.^9^ We also found that G2/M and S phases arrest significantly promoted replication of SARS-CoV-2. Coronaviruses have used cell cycle regulation to participate in the pathogenicity of viral infection.^10^ However, the G2/M and S phases arrest related to pathogenesis of SARS-CoV-2 need further investigation.

The expression of cyclin B1, CDK1 and CDK2 are important for G2/M and S progression. Our results showed that the nuclear translocation of cyclin B1 and CDKs was decreased upon SARS-CoV-2 infection, which may be partly responsible for the G2/M phase arrest during SARS-CoV-2 infection. The phosphorylation level of T14/Y15 on CDK1, related to its activation, has been demonstrated to be dysregulated after SARS-CoV-2 infection.^9^ WEE1 is the main kinase that phosphorylates CDK1 at Y15, and CDC25C dephosphorylates CDK1 at T14 and Y15.^11^ In the LC-MS analysis, the expression of WEE1 was significantly increased at 12 hpi and decreased at 24 hpi (Supplemental Fig. S3), while the abundance of CDC25C showed no significant change, indicating that WEE1 may be involved in the dysregulated phosphorylation status of CDK1 upon SARS-CoV-2 infection.

Viruses regulate the activity of APC/C, which can inhibit host cells exit mitosis and arrest at the G2/M phase.^12,13^ The adenovirus E4orf4 protein elicits cell cycle block at the G2/M phase by altering the activity of APC/C.^14^ Human papillomaviruses (HPVs) from the high-risk group interact with Cdc20 and Cdh1, the two regulators of APC/C, to promote a mitotic block.^15^ The SAC is also a regulator of APC/C and can temporarily delay anaphase processes to ensure that all kinetochores are connected to the mitotic spindle.^16^ AURKA is a substrate of APC/C related to spindle formation and mitotic arrest.^17^ Our study indicated that most of the substrates of APC/C were increased upon SARS-CoV-2 infection, and the expression of Cdc20, AURKA and the SAC members Bub1 and Bub3 were also dysregulated after SARS-CoV-2 infection, which may relate to the G2/M phase arrest of viral infection. SARS-CoV-2 infection was prone to induce S phase arrest at early infection stage, and to induce G2/M phase arrest at the late infection stage, which may lead to up-regulation of Bub1, Cdc20 and AURKA at 48 hpi. For the four decreased substrates of APC/C, OPA-1 and TFAM (Transcription factor A) function in mitochondrial morphology and transcription regulation. ^18,19^ MAD2L1 and MAD2L2 are mitotic spindle assembly checkpoint proteins, which could inhibit Cdc20-mediated activation of APC/C,^20,21^ the decreased MAD2L1 and MAD2L2 may antagonize SARS-CoV-2 induced suppression of APC/C.

Several coronaviruses have been demonstrated to manipulate the activity of cyclin-CDK complex and P53 signaling to induce cell cycle arrest at the S and G2/M phases.^22^ Porcine epidemic diarrhea virus N protein induces S phase arrest via interacting with P53.^23^ TGEV N protein induces G2/M and S phases arrest by activating P53 signaling.^24^ SARS-CoV N protein has been demonstrated to block S phase progression by inhibiting activity of cyclin-CDK complex.^25^ SARS-CoV-2 infection also manipulated P53 signaling in the LC-MS analysis, which may be utilized by SARS-CoV-2 to induce cell cycle arrest at the S and G2/M phases.

Viral proteins can directly interact with cell cycle related host proteins to regulate cell cycle progression. ^7^ When we mapped significantly up- or down-regulated host proteins to the interactome of SARS-CoV-2 ^5^, host proteins were shown to correlate with the accessory proteins of orf3a, orf8, orf9b, orf9c and orf10, and nonstructural proteins of nsp2, nsp8, nsp12 and nsp13, respectively (Supplemental Fig. S8 and Table S2 and S3). Among the 13 host-viral protein interactions, nsp13 interacted with the centromeric protein F (CENPF), which is a mitotic centromere protein involved in chromosome segregation during mitosis of the cell cycle.^26^ CENPF is a substrate of APC/C that has been reported to interact with CDK1 to regulate G2/M transition,^27^ with a similar expression pattern of CDK1 upon SARS-CoV-2 infection (Supplemental Fig. S6a). In addition, the nuclear membrane protein SUN2 interacts with orf3a, which was decreased during the viral infection. SUN2 is essential for the nuclear envelope breakdown during the early phase of mitosis, and depletion of SUN2 results in an accumulation of morphologically defective and disoriented mitotic spindles, thus delaying the mitosis progression.^28^

Reversine is a pan inhibitor of aurora kinases to inhibit the SAC.^29^ Reversine can reverse the mitotic arrest induced by HPV E6 and E7,^30^ and inhibit the replication of Epstein-Barr virus.^31^ MK5108 is in phase I clinical trial for patients with advanced or refractory solid tumors ^32^ and AT9283 is in phase II clinical trial for multiple solid tumors.^33^ AT9283 also inhibits the activity of JAK2, which may be associated with its higher antiviral effects as comparison with MK5108. The antiviral activity of these compounds suggests that SAC and AURKA may become potential drug targets against SARS-CoV-2 infection. The dysregulated proteins including AURKA, Cdc20, and AURKB were identified as potential drug targets for COVID-19 in a bioinformatics and machine learning analysis.^34,35^

In summary, our findings reveal that SARS-CoV-2 manipulates cell cycle checkpoint and induces host cells arrest at the G2/M and S phases to facilitate viral replication, while inhibitors of SAC and AURKA can effectively inhibit viral replication, suggesting a potential antiviral target of host cell cycle checkpoint for COVID-19.

**Referances**

1 Parkhomchuk, D. et al. Transcriptome analysis by strand-specific sequencing of complementary DNA. *Nucleic Acids Res* **37**, e123 (2009).

2 Sui, L. et al. SARS-CoV-2 membrane protein inhibits type I interferon production through ubiquitin-mediated degradation of TBK1. *Front Immunol* **12**, 662989 (2021).

3 Zhao, Y. et al. A dual-role of SARS-CoV-2 nucleocapsid protein in regulating innate immune response. *Signal Transduct Target Ther* **6**, 331 (2021).

4 Bojkova, D. et al. Proteomics of SARS-CoV-2-infected host cells reveals therapy targets. *Nature* **583**, 469-472 (2020).

5 Gordon, D. E. et al. A SARS-CoV-2 protein interaction map reveals targets for drug repurposing. *Nature* **583**, 459-468 (2020).

6 Wang, Z. Y. et al. Human Enterovirus 68 Interferes with the Host Cell Cycle to Facilitate Viral Production. *Front Cell Infect Microbiol* **7**, 29 (2017).

7 Liu, S. et al. The severe fever with thrombocytopenia syndrome virus NSs protein interacts with CDK1 to induce G2cell cycle arrest and positively regulate viral replication. *J Virol* **94**, e01575-01519 (2020).

8 Gearhart, T. L. & Bouchard, M. J. Replication of the hepatitis B virus requires a calcium-dependent HBx-induced G1 phase arrest of hepatocytes. *Virology* **407**, 14-25 (2010).

9 Bouhaddou, M. et al. The Global Phosphorylation Landscape of SARS-CoV-2 Infection. *Cell* **182**, 685-712.e619 (2020).

10 Chau, T. N. et al. SARS-associated viral hepatitis caused by a novel coronavirus: report of three cases. *Hepatology* **39**, 302-310 (2004).

11 Davy, C. & Doorbar, J. G2/M cell cycle arrest in the life cycle of viruses. *Virology* **368**, 219-226 (2007).

12 Bagga, S. & Bouchard, M. J. Cell cycle regulation during viral infection. *Methods Mol. Biol.* **1170**, 165-227 (2014).

13 Mo, M., Shahar, S., Fleming, S. B. & Mercer, A. A. How viruses affect the cell cycle through manipulation of the APC/C. *Trends Microbiol.* **20**, 440-448 (2012).

14 Mui, M. Z. et al. Adenovirus protein E4orf4 induces premature APCCdc20 activation in Saccharomyces cerevisiae by a protein phosphatase 2A-dependent mechanism. *J Virol* **84**, 4798-4809 (2010).

15 Bellanger, S., Blachon, S., Mechali, F., Bonne-Andrea, C. & Thierry, F. High-risk but not low-risk HPV E2 proteins bind to the APC activators Cdh1 and Cdc20 and cause genomic instability. *Cell Cycle* **4**, 1608-1615 (2005).

16 Musacchio, A. Spindle assembly checkpoint: the third decade. *Philos. Trans. R. Soc. Lond. B Biol. Sci.* **366**, 3595-3604 (2011).

17 Cowley, D. O. et al. Aurora-A kinase is essential for bipolar spindle formation and early development. *Mol Cell Biol* **29**, 1059-1071 (2009).

18 Ishihara, N., Fujita, Y., Oka, T. & Mihara, K. Regulation of mitochondrial morphology through proteolytic cleavage of OPA1. *Embo j* **25**, 2966-2977 (2006).

19 Celestini, V. et al. Uncoupling FoxO3A mitochondrial and nuclear functions in cancer cells undergoing metabolic stress and chemotherapy. *Cell Death Dis.* **9**, 231 (2018).

20 Iwai, H. et al. A bacterial effector targets Mad2L2, an APC inhibitor, to modulate host cell cycling. *Cell* **130**, 611-623 (2007).

21 Luo, X., Tang, Z., Rizo, J. & Yu, H. The Mad2 spindle checkpoint protein undergoes similar major conformational changes upon binding to either Mad1 or Cdc20. *Mol Cell* **9**, 59-71 (2002).

22 Su, M. et al. A mini-review on cell cycle regulation of coronavirus infection. *Front Vet Sci* **7**, 586826 (2020).

23 Su, M. et al. Coronavirus porcine epidemic diarrhea virus nucleocapsid protein interacts with p53 to induce cell cycle arrest in S-phase and promotes viral replication. *J Virol* **95**, e0018721 (2021).

24 Ding, L. et al. TGEV nucleocapsid protein induces cell cycle arrest and apoptosis through activation of p53 signaling. *Biochem Biophys Res Commun* **445**, 497-503 (2014).

25 Surjit, M., Liu, B., Chow, V. T. & Lal, S. K. The nucleocapsid protein of severe acute respiratory syndrome-coronavirus inhibits the activity of cyclin-cyclin-dependent kinase complex and blocks S phase progression in mammalian cells. *J Biol Chem* **281**, 10669-10681 (2006).

26 Berto, A. & Doye, V. Regulation of Cenp-F localization to nuclear pores and kinetochores. *Cell Cycle* **17**, 2122-2133 (2018).

27 Huang, Y.-g., Li, D., Wang, L., Su, X.-m. & Tang, X.-b. CENPF/CDK1 signaling pathway enhances the progression of adrenocortical carcinoma by regulating the G2/M-phase cell cycle. *J. Transl. Med.* **20**, 78 (2022).

28 Turgay, Y. et al. SUN proteins facilitate the removal of membranes from chromatin during nuclear envelope breakdown. *J. Cell Biol.* **204**, 1099-1109 (2014).

29 Santaguida, S., Tighe, A., D'Alise, A. M., Taylor, S. S. & Musacchio, A. Dissecting the role of MPS1 in chromosome biorientation and the spindle checkpoint through the small molecule inhibitor reversine. *J. Cell Biol.* **190**, 73-87 (2010).

30 Hayashi, M. T., Cesare, A. J., Rivera, T. & Karlseder, J. Cell death during crisis is mediated by mitotic telomere deprotection. *Nature* **522**, 492-496 (2015).

31 Ling, P. D. et al. Phosphoproteomic profiling reveals Epstein-Barr virus protein kinase integration of DNA damage response and mitotic signaling. *PLoS Pathog.* **11**, e1005346 (2015).

32 Amin, M. et al. A phase I study of MK-5108, an oral aurora a kinase inhibitor, administered both as monotherapy and in combination with docetaxel, in patients with advanced or refractory solid tumors. *Invest. New Drugs* **34**, 84-95 (2016).

33 Mazzera, L. et al. Aurora and IKK kinases cooperatively interact to protect multiple myeloma cells from Apo2L/TRAIL. *Blood* **122**, 2641-2653 (2013).

34 Auwul, M. R., Rahman, M. R., Gov, E., Shahjaman, M. & Moni, M. A. Bioinformatics and machine learning approach identifies potential drug targets and pathways in COVID-19. *Brief. Bioinform.* **22**, bbab120 (2021).

35 Yang, L. et al. Network pharmacology and comparative transcriptome reveals biotargets and mechanisms of curcumol treating lung adenocarcinoma patients with COVID-19. *Front Nutr* **9**, 870370 (2022).
